# Inhibition of mTOR Reduces Anal Carcinogenesis in Transgenic Mouse Model

**DOI:** 10.1371/journal.pone.0074888

**Published:** 2013-10-04

**Authors:** Zhi-Jun Sun, Lu Zhang, Wei Zhang, Bradford Hall, Yansong Bian, Ashok B. Kulkarni

**Affiliations:** 1 The State Key Laboratory Breeding Base of Basic Science of Stomatology & Key Laboratory of Oral Biomedicine Ministry of Education, School and Hospital of Stomatology, Wuhan University, Wuhan, China; 2 Functional Genomics Section, Laboratory of Cell and Developmental Biology, National Institute of Dental and Craniofacial Research, National Institutes of Health, Bethesda, Maryland, United States of America; 3 Head and Neck Surgery Branch, National Institute of Deafness and Other Communicative Disorders, National Institutes of Health, Bethesda, Maryland, United States of America; Peter MacCallum Cancer Centre, Australia

## Abstract

The molecular mechanism of human anal squamous cell carcinoma (ASCC) is unclear, and the accumulating evidence indicate association of ASCC with the activation of the Akt/mTOR pathway. Here we describe a mouse model with spontaneous anal squamous cell cancer, wherein a combined deletion of *Tgfbr1* and *Pten* in stratified squamous epithelia was induced using inducible K14-Cre. Histopathologic analyses confirmed that 33.3% of the mice showed increased susceptibility to ASCC and precancerous lesions. Biomarker analyses demonstrated that the activation of the Akt pathway in ASCC of the *Tgfbr1* and *Pten* double knockout (2cKO) mouse was similar to that observed in human anal cancer. Chemopreventive experiments using mTOR inhibitor-rapamycin treatment significantly delayed the onset of the ASCC tumors and reduced the tumor burden in 2cKO mice by decreasing the phosphorylation of Akt and S6. This is the first conditional knockout mouse model used for investigating the contributions of viral and cellular factors in anal carcinogenesis without carcinogen-mediated induction, and it would provide a platform for assessing new therapeutic modalities for treating and/or preventing this type of cancer.

## Introduction

Anal cancer is an uncommon malignancy located in the anal canal and perianal area, with an annual incidence of 1.5 per 100,000 in the general population [1], [2]. The incidence of anal cancer in the United States has been rising over the past three decades, especially in some subpopulations; for example, homosexual men are at a higher risk for anal cancer [1], [2]. The 5-year survival rate for those suffering from anal cancer has remained consistently low and nearly unchanged, at approximately 60% over the past 30 years [1]. Etiologically, anal cancer seems to be more similar to genital cancers than to gastrointestinal tract cancers. Like cervical cancer, the human papillomavirus (HPV) infection is considered to be an important etiological factor in the development of ASCC due to the high rate of HPV infection in patients with anal cancer [3−5]. However, the HPV oncogenes which lead to increases in cell proliferation and evasion from the apoptotic pathway are considered insufficient for causing this tumor [6]. Another important molecular change that has been reported in 66% of anal cancer cases is the cellular accumulation of phosphorylated Akt and the subsequent nuclear translocation of TP53 [7]. The increased phosphorylated-Akt may be due to increased copy numbers of the PIK3CA locus and some coding sequence mutations or HPV infection [7], [8]. PTEN is a potent, tumor suppressor gene and a negative regulator of the PI3K/Akt pathway [9]. TGF-β belongs to a superfamily of multifunctional cytokines that regulate cell apoptosis, differentiation, and migration, thereby influencing the key physiological processes such as embryonic development, immune function, and carcinogenesis [10]. The three mammalian TGF-β isoforms, TGF-β1, -β2, and -β3 exert their functions through a cell-surface receptor complex composed of type I (TGFBR1) and type II (TGFBR2) serine/threonine kinase receptors [11]. We previously reported that the deletion of the TGF-β receptor I (Tgfbr1) promotes tumorigenesis of head and neck squamous cell carcinoma, mainly through the activation of the Akt pathway, but it does not initiate it [12]. The loss of Pten alone in the squamous epithelia can initiate the mouse squamous cell tumorigenesis with about 10% penetration [13].

In order to better understand the mechanism of anal cancer and to identify novel therapeutic approaches for preventing and/or treating the malignancy, laboratory animal models for anal cancer were established to provide an experimental platform. Lambert's lab developed a murine anal cancer model using HPV E6/E7 transgenic mice, in which the E6 and E7 genes are linked to the K14 promoter targeting their expression to stratified epithelium [6], [14]. This model greatly promotes our understanding of the molecular mechanism of anal cancer and provides a preclinical platform to test the effects of the novel drug in anal cancer treatment [6], [14]. However, these HPV transgenic mice do not spontaneously develop anal cancer and must be treated with carcinogen, dimethylbenzanthracene (DMBA), or 12-O- tetradecanoylphorbol-13-acetate (TPA). We previously developed *Tgfbr1* conditional knockout mice with Neurofilament-H- Cre, which develop anal cancer over a long period of about 4–6 months [15]. Our previous study also suggested that there may be a negative cross talk between the TGF-β tumor suppressor and the PI3K/Akt pathways [12]. Here we report that *Tgfbr1/Pten* double conditional knockout mice spontaneously develop anal cancer in a short period of time with activation of the Akt/mTOR pathway and without carcinogen induction. We have also have identified therapeutic effects of rapamycin, a putative mTOR inhibitor, which can inhibit tumorigenesis of ASCC in this mouse model.

## Materials and Methods

### Mice

Generation of *Tgfbr1/Pten* 2cKO (K14-CreER^tam^; *Tgfbr1* ^flox/flox^; *Pten* ^flox/flox^) mice, *Tgfbr1* cKO mice (K14-CreER^tam^; *Tgfbr1* ^flox/flox^) and *Pten* cKO (K14-CreER^tam^; *Pten* ^flox/flox^) mice has been previously described [12], [13], [16], [17]. The *Tgfbr1/Pten* 2cKO mice and their controls (*Tgfbr1* ^flox/flox^; *Pten* ^flox/flox^) were from the same litter, with a mixed genetic background of C57BL/6; FVBN; CD1; 129. Mice were housed in a controlled environment and all animal procedures were performed in compliance with the NIH guidelines for the Care and Use of Laboratory Animals and approved by IACUC, NIDCR (Permit Number 09-530). One- to two-month-old male and female mice received a tamoxifen-mediated induction procedure that has been previously described [12], [13], [16], [17].

### Rapamycin treatment

Rapamycin (LC Laboratory, Woburn, MA) was dissolved as previously described [16]. Two weeks after the last oral dose of tamoxifen, the mice were randomized into a control group (n = 17 mice) or a group that received 10 mg/kg rapamycin i.p. every other day (n = 17 mice). Mice were treated with this rapamycin dosing schedule for 6 weeks, and tumor size was measured weekly. Tumor volume was calculated by multiplying the three dimensions of each tumor using a micrometer caliper. Tumor burden was calculated as the individual tumor volume in each mouse and normalized with relative tumor growth by dividing the final volume by the initial tumor volume. At the end of the tumorigenesis studies, the mice were euthanized using CO_2_; tissues were harvested and then fixed in buffered zinc formalin (Z-fix, Anatech, Battle Creek, MI, USA) overnight. The tissues were then transferred to 70% alcohol and processed for paraffin embedding for a histopathological diagnosis and further studies.

### Histology and Immunohistochemistry

To determine tumor multiplicity of the anal tissue, the tumor invasive depth and tumor size were counted using an Aperio CS scanscope digital imaging system [16]. Antibodies against PI3K p110α(1:400), p-Akt (S473,1∶50), p-S6 (S235/236, 1∶200) were purchased from Cell Signaling Technology (Danvers, MA, USA), and mouse Ki-67 (1∶400), were purchased from DAKO (Carpinteria, CA,USA). The sections of the *Tgfbr1/Pten* 2cKO anal SCC samples (n = 5), as compared with *Tgfbr1/Pten* 2cKO anal skin (n = 5), *Tgfbr1^flox/flox^/Pten^flox/flox^* anal skin (n = 5), and rapamycin treated *Tgfbr1/Pten* 2cKO anal skin (n = 3) were stained with the antibody by immunohistochemistry using an appropriate biotin-conjugated, secondary antibody and a Vectastain ABC Elite kit (Vector Laboratories, Burlingame, CA), as previously reported in protocols [16]. Slices were scanned using an Aperio ScanScope CS scanner (Vista, CA) with background substrate for each slice, and quantified using Aperio Quantification software (Version 9.1) for membrane, nuclear, or pixel quantification. An area of interest was selected in either the epithelial or the cancerous area for scanning and quantification. Histoscore was calculated as previously described [16]. Briefly, four random high power field (20×) of each slides with membrane and nuclear immunostaining was calculated as a percentage of different positive cells using the formula (3+)×3+(2+)×2+(1+)×1. Histoscore of pixel quantification was calculated as total intensity/total cell number. The threshold for scanning of different positive cells was set by a pathologist according to the standard controls provided by Aperio.

### Deoxynucleotidyl transferase-mediated dUTP nick end labeling (TUNEL) assay

Apoptotic cells in tumor tissues were quantified by the terminal deoxynucleotidyl transferase-mediated dUTP nick end labeling (TUNEL) assay as previous described [16]. TUNEL staining was performed using the in situ cell death detection kit, POD (Roche, Mannheim, Germany) according to the manufacturer's instructions. Then six representative high power field areas (20×) of each section of vehicle treated (n = 5) and rapamycin treated group (n = 3) without necrosis were selected and both apoptotic cells and total cells were counted under a light microscope.

### Western blot analysis

Harvested tissues were lysed in T-PER (Pierce, Rockford, IL) containing a complete mini-protease inhibitor cocktail and phosphate inhibitors (Roche, Branchburg, NJ). Skin around anal area was harvested from two individual *Tgfbr1^flox/flox^/Pten^flox/flox^*mice and two *Tgfbr1/Pten* 2cKO mice, and three tumors harvested from *Tgfbr1/Pten* 2cKO mice were used for Western blot analysis. Antibodies against PI3K p110α, PI3K p85, p-Akt (S473), Akt, p-S6 (S235/236), S6, p-P70S6K (T389), P70S6K, p-Stat3(T705), p-4E-BP1(S65), and survivin were purchased from Cell Signaling Technology (Danvers, MA,USA). Detailed procedures for immunoblotting performed were as described previously [12], [13], [16], [17].

### RNA isolation and quantitative reverse transcription-PCR

Total RNAs were extracted from anal tumor of the *Tgfbr1/Pten* 2cKO mice treated with rapamycin or vehicle only group (3 tumors from each group) using miRNeasy kit (Qiagen, Valencia, CA). First-strand cDNAs were synthesized using the Superscript III Reverse Transcription kit (Invitrogen, Carlsbad, CA). Quantitative reverse transcription-PCR analysis using iQ SYBR Green Supermix was done by the absolute standard curve method using the Chrom 4 Real-time PCR System (BioRad, Hercules, CA) as previous described [12], [13], [16]. For the detection of relative mRNA level in tumors and skin, RT-PCR was performed using QuantiTect Primers for mouse Ccl2, Ccl3, Cxcl1, Cxcl5, Il1a, Il1b, Tnf, Ptgs1, Akt1, Rps6 and Mki67 (Qiagen, Valencia, CA). The mean values of relative mRNA level were based on triplicate wells from 3 independent experiments.

### Statistical analysis

Data analyses were done using Graph Pad Prism version 5.0 for Windows (Graph-Pad Software Inc, La Jolla, CA). One-way ANOVA followed by the post-Turkey or Dunnett multiple comparison tests were used to analyze the differences in immunostaining, protein levels and relative mRNA level among each group or as compared with control group. The Mann – Whitney *U* test and Student *t* test was used to evaluate differences between the total tumor areas of the mice treated with rapamycin as compared to untreated mice. Values of *P*<0.05 were considered statistically significant.

## Results

### Anal Carcinogenesis in Tgfbr1/Pten 2cKO mice

**Figure 1:**
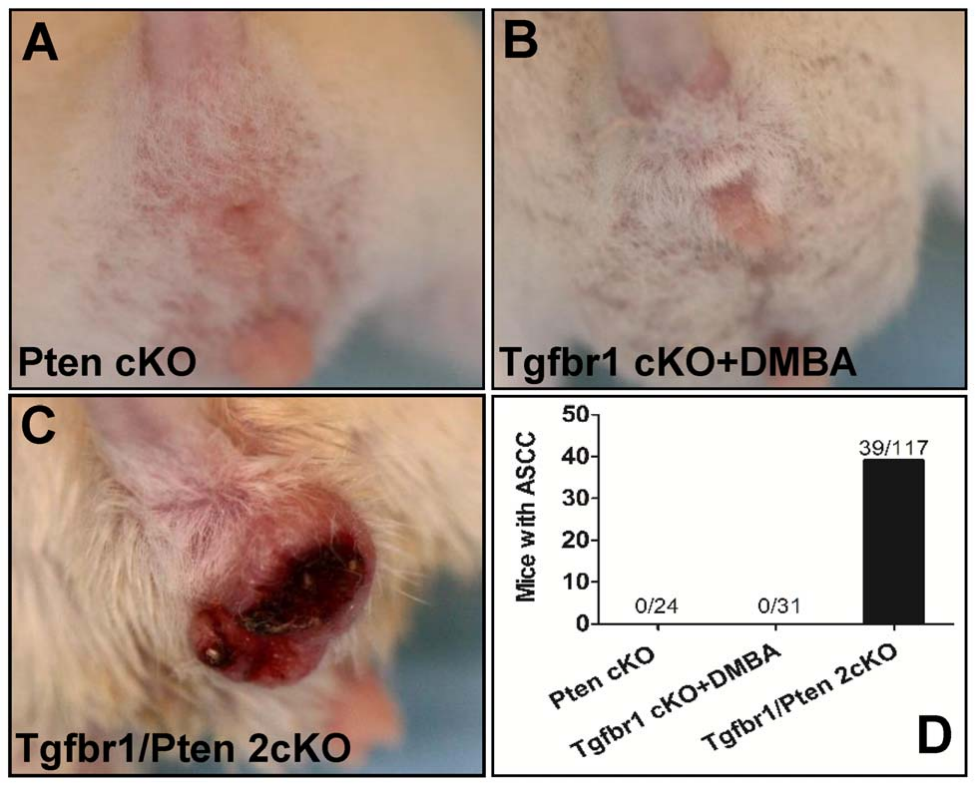
Anal squamous cell carcinoma in *Pten* cKO mice, *Tgfbr1* cKO mice and *Tgfbr1/Pten* 2cKO mice. ASCC tumors were not observed in *Pten* cKO mice (A), *Tgfbr1* cKO mice (B). ASCC tumor was located in the anal canal and perianal skin area of *Tgfbr1/Pten* 2cKO mice. (D) The frequency of ASCC tumors observed in *Pten* cKO, *Tgfbr1* cKO and *Tgfbr1/Pten* 2cKO mice.

**Figure 2:**
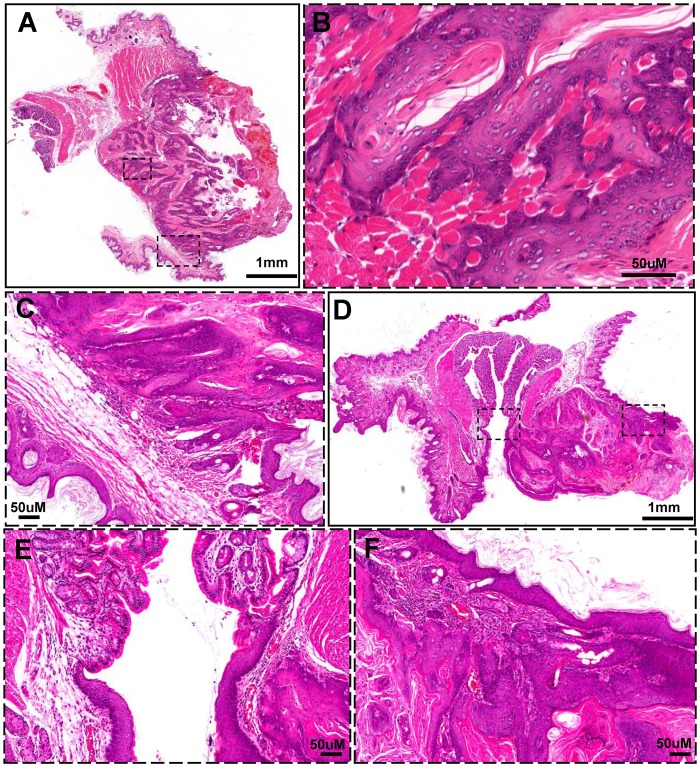
Histological feature of ASCC in *Tgfbr1/Pten* 2cKO mice. (A), Representative saggital sections of ASCC tumors showing differentiated epithelial cells that grew as solid sheets or nests. Invasion of ASCC into surrounding tissue (B) and origination of ASCC (C). Coronal section of ASCC showing ASCC tumor in squamous epithelial (D) but not in columnar epithelial cells (E, F). (Hematoxylin and eosin staining).

*Pten* cKO mice (Fig 1A) and *Tgfbr1* cKO mice (even with one dose of 50 mg DMBA, Fig 1B) did not develop anal cancer during observation of more than one year. Interestingly, 4 weeks after oral administration of tamoxifen for 5 consecutive days, hyperplasia was observed in the perianal areas of the *Tgfbr1/Pten* 2cKO mice. The visible anal tumors were observed in 6 weeks (42±14 days, Figs. 1C and 1D) in 39 out of 117 (33.3%) mice based on 16 weeks observation after tamoxifen induction. All the 2cKO mice were euthanized 16 weeks after tamoxifen induction because they developed HNSCC tumors of the size exceeding the limit set by NIH guidelines. Representative histopathological images of anal neoplasm arising in the *Tgfbr1/Pten* 2cKO mice are shown in Figs. 2. The tumor originated from squamous epithelial but not from columnar epithelial (Figs 2Aand 2B), which is composed of atypical differentiated epithelial cells that grew as solid sheets or strands, a hallmark feature of squamous cell carcinoma Fig. 2B. The invasion into the adjacent muscle tissues was observed in several tumor samples, indicating the aggressive nature of the tumor (Fig. 2 B).

### Activation of Akt, mTOR, and increased proliferation in Tgfbr1/Pten 2cKO mouse anal SCC

**Figure 3:**
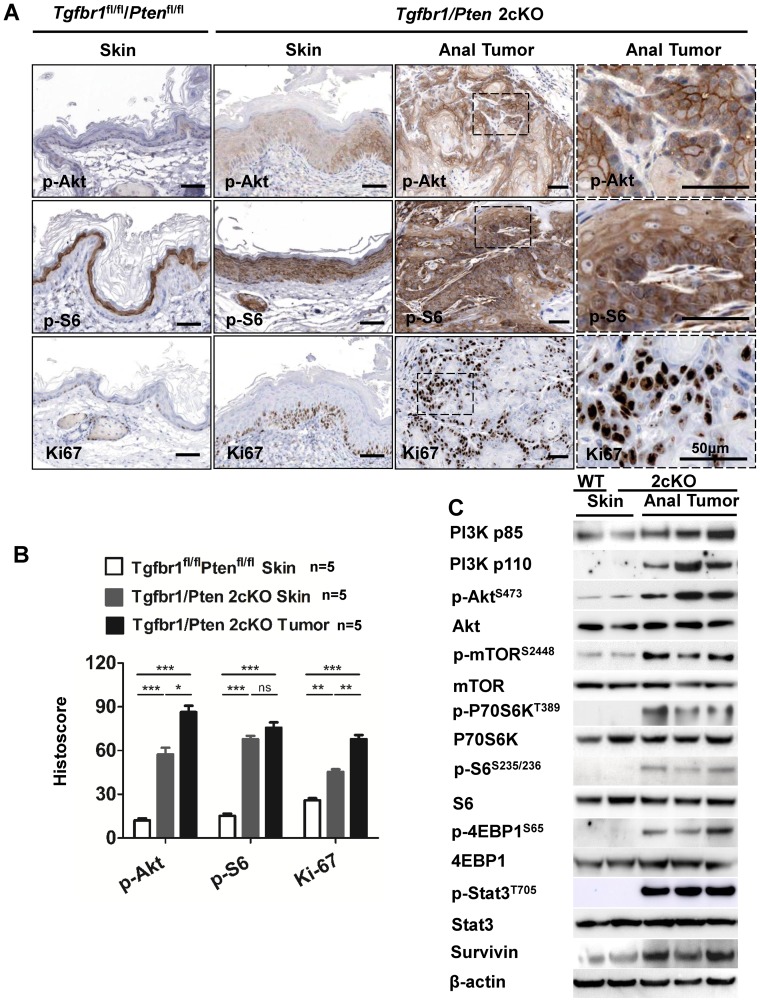
Activation of Akt/p-S6/Ki67 in *Tgfbr1/Pten* 2cKO ASCC. (A), immunostaining with specific antibodies indicates increased expression of p-Akt, p-S6, and Ki67 in *Tgfbr1/Pten* 2cKO mice ASCC (higher magnification is shown in the right most cloumn) as compared with *Tgfbr1^flox/flox^/Pten^flox/flox^* skin (scale bars  = 50 μm). (B), the quantification of immunostaining shown in panel (A) presented as histoscore. Mean ± SEM; *, *P*<0.05; **, *P*<0.01; ***, *P*<0.001: One-way ANOVA. (C), Western blot analysis shows a significant increase in p-Akt, p-mTOR, p-P70S6K, p-S6, p-4E-BP1, and survivin in *Tgfbr1/Pten* 2cKO ASCC as compared with *Tgfbr1^flox/flox^/Pten^flox/flox^* anal skin. The total protein of Akt, P0S6K and S6 remain unchanged.

Since mTOR signaling pathways were activated in *Tgfbr1/Pten* 2cKO mouse HNSCC as reported previously, it would be interesting to investigate whether these pathways were similarly activated in the anal SCC. Immunostaining of p-Akt, p-S6, and Ki67 in the tumors was performed. Our results revealed intense staining for the phosphorylated form of Akt (p-Akt^S473^), and for p-serine ribosomal protein S6 (p-S6 ^S235/236^) (Fig 3A) in the *Tgfbr/Pten* 2cKO anal SCC tissue section, as compared with *Tgfbr1^flox/flox^/Pten^f flox/flox^* mouse perianal skin (Figs. 3A and B). Increased staining for Ki67, a hallmark of proliferation, was also observed in 2cKO mouse anal tumors as compared with *Tgfbr1/Pten* 2cKO perianal skin (*P*<0.01) and *Tgfbr1^flox/flox^/Pten^flox/flox^* perianal skin (*P*<0.01, Figs. 3A and B). Most importantly, the marked increase of p-mTOR^S2448^ coincided with the increased levels of PI3K p110, PI3K p85, p-Akt^S473^, p-P70S6K^T389^, p-S6^S235/236^, p-Stat3^T705^ and p-4E-BP1^S65^ in 2cKO tumors (Fig. 3C), while the total protein levels of Akt, P70S6K and S6 remained unchanged (Fig. 3C). The protein levels of survivin, a downstream target of mTOR, also increased significantly in ASCC (Fig. 3C).

### Increased levels of pro-inflammatory cytokines in Tgfbr1/Pten 2cKO mouse anal SCC

**Figure 4:**
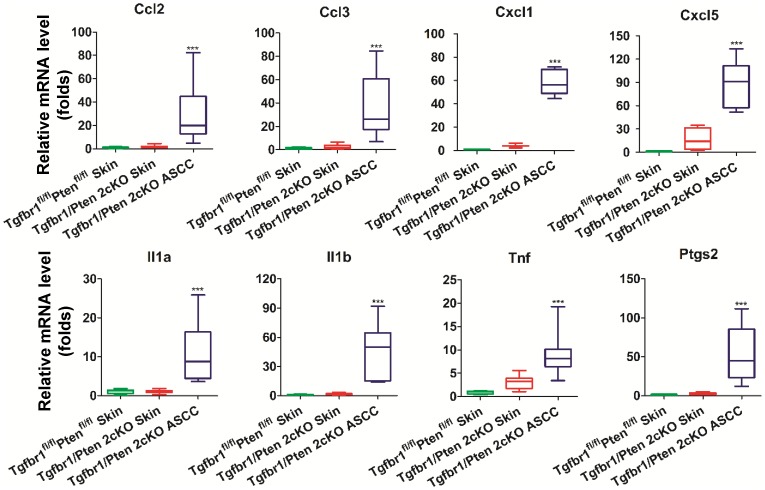
Increased levels of pro-inflammatory cytokine in *Tgfbr1/Pten* 2cKO mice ASCC. Real-time PCR analysis shows increased expression of Ccl2, Ccl3,Cxcl1,Cxcl5,Il1a,IL1b,Tnf,Ptgs2 in *Tgfbr1/Pten* 2cKO mice ASCC compared with *Tgfbr1^flox/flox^/Pten^flox/flox^* anal skin and *Tgfbr1/Pten* 2cKO anal skin. n = 5 in each group, Mean ± SEM; ***, *P*<0.001: One-way ANOVA with post-Dunnett test.

Since the pro-inflammatory cytokine levels correlate with anal tumorigenesis, it is reasonable to investigate whether these proteins were activated similarly in the anal SCC. Real-time PCR shows an increase of Ccl2, Ccl3, Cxcl1, Cxcl5, Il1a, IL1b, Tnf, Ptgs2 (Fig 4, all *P*<0.001) in the *Tgfbr/Pten* 2cKO anal SCC tissue section, as compared with *Tgfbr1^flox/flox^/Pten^f flox/flox^* mouse perianal skin.

### Rapamycin treatment delays the onset, and reduces progression of tumorigenesis in Tgfbr/Pten 2cKO mouse anal SCC

**Figure 5:**
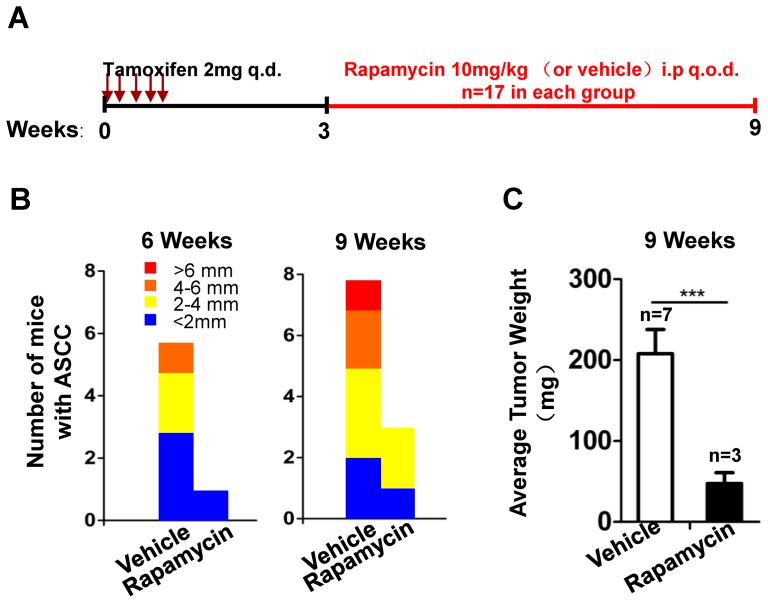
Inhibition of mTOR by rapamycin treatment blocks ASCC progression in *Tgfbr1/Pten* 2cKO mice. (A), a schematic showing a rapamycin treatment used in the chemopreventive experiment in *Tgfbr1/Pten* 2cKO mice. (B), reduced tumor burden in *Tgfbr1/Pten* 2cKO mice by rapamycin treatment as compared with vehicle group 6 weeks (left panel) and 9 weeks (right panel) after tamoxifen induction, respectively The total tumor number of tumors observed is calculated from 17 mice in each group. (C), average ASCC tumor weight in rapamycin- and vehicle- treated groups at 9 week is calculated using Graph pad Prisim5. Mean ± SEM; *, *P*<0.05; **, *P*<0.01; ***, *P*<0.001; Student *t* test. i.p., intra-peritoneal, q.o.d., every other day; rapamycin group, n = 17, vehicle group, n = 17.

We previously reported that both Tgfbr1 and Pten deletion increased the phosphorylation levels of mTOR and its downstream target in *Tgfbr1* cKO or *Pten* cKO mouse HNSCC [13]. Therefore, we determined whether rapamycin, the specific inhibitor of mTOR, could halt or delay tumorigenesis in *Tgfbr1/Pten* 2cKO mouse anal SCC. Rapamycin treatment (10 mg/kg, i.p, q.o.d) was carried out 2 weeks after tamoxifen administration for 5 consecutive days, inducing Cre-mediated deletion of *Tgfbr1* and *Pten* in the mouse (Fig. 5A). Rapamycin treatment significantly delayed and reduced the progression of squamous cell carcinoma in the perianal area at 6 and 9 weeks after the start of treatment. The incidence (6/17 versus 1/17 in 6 weeks, and 8/17 versus 3/17 in 9 weeks) and tumor volume were remarkably reduced after rapamycin treatment (Fig. 5B). The tumor burden of the mice that received rapamycin treatment was much lower after 9 weeks than the tumor burden of the mice that received the vehicle treatment (*P*<0.05,Fig. 5C).

### Rapamycin treatment decreases cell proliferation and Akt/mTOR signaling in Tgfbr1/Pten 2cKO mice anal SCC

**Figure 6:**
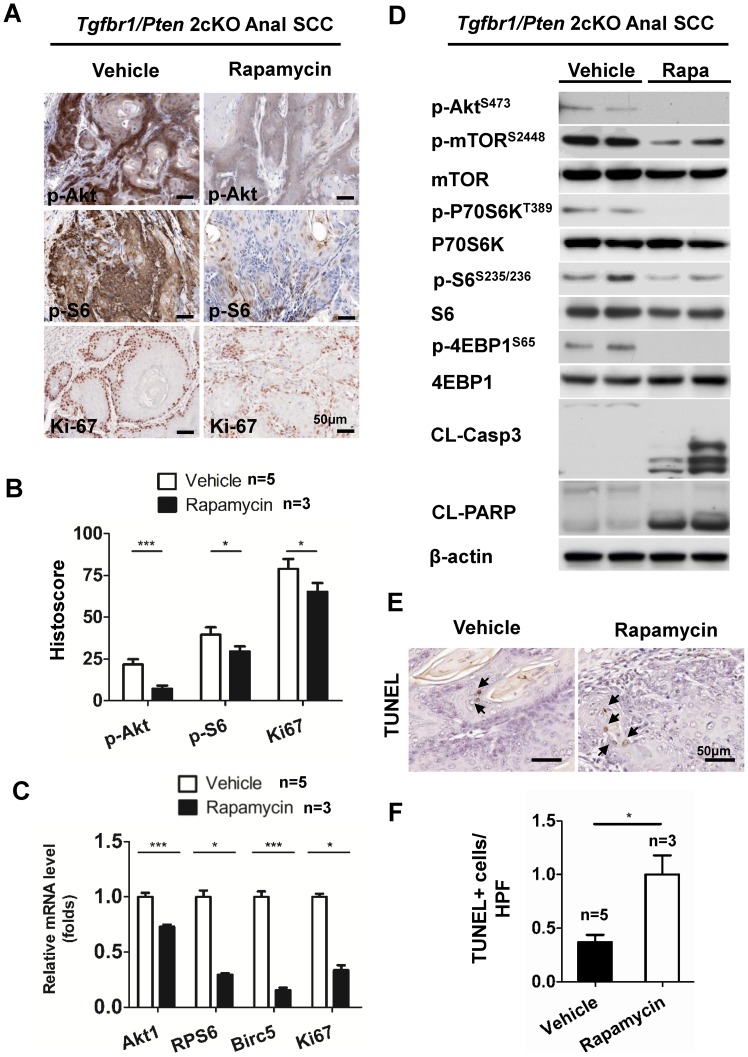
Decrease in Akt/mTOR signaling and cell proliferation in *Tgfbr1/Pten* 2cKO ASCC after rapamycin treatment. (A), representative immunostaining for p-Akt, p-S6, and Ki67 in vehicle- and rapamycin-treated groups. (B), quantification of staining by histoscores shows a significant decrease in p-Akt, p-S6, and Ki67. (C), Western blot analysis shows significant decrease in the levels of p-Akt, p-mTOR, p-P70S6K, p-S6, and p-4E-BP1 but not in the Akt, P70S6k and S6. Rapamycin treatment increased protein levels of cleaved caspase-3 (CL-Casp3) and cleaved PARP (CL-PARP). Rapa; Rapamycin, (D), Quantification for relative mRNA levels of Akt1, Rps6, Birc5 and Mki67 shows decreased mRNA levels in rapamycin-treated group (n = 3) as compared with vehicle-treatedgroup(n = 5). (E), increased apoptotic cells were detected by TUNEL in rapamycin- treated group (n = 3) as compared with vehicle-treated group (n = 5). (F), quantification of TUNEL positive cells per high power field of 20×shows increased apoptosis after rapamycin treatment. Mean ± SEM; *, *P*<0.05; ***, *P*<0.001; Student *t* test.

In order to analyze the underlying mechanism that caused rapamycin to suppress tumorigenesis in the *Tgfbr1/Pten* 2cKO mouse, we performed immunostaining for the Akt/mTOR signaling pathway, Ki67 for proliferation, and TUNEL for apoptosis in the rapamycin treatment mice. The rapamycin treatment significantly decreased the staining intensities of p-Akt^S473^ and p-S6^S235/236^ in the *Tgfbr1/Pten* 2cKO mouse (Figs. 6A and B). This is further confirmed by the decreased mRNA levels of Akt1, Rps6, Birc5, and Mki67, which correlated with the mTOR pathway in rapamycin-treated mice ASCC (n = 5) as compared with vehicle group (n = 5, *P*<0.001, Fig. 6D). The results from Western blots also confirmed the decreased phosphorylation of Akt, mTOR, P70S6k, S6, and 4E-BP1 but increase in cleaved caspase-3 and cleaved PARP in rapamycin-treated mice tumor (Fig. 6 C), The suppressed expression of Ki67 in rapamycin-treated mice displayed the inhibitory effects of rapamycin in tumor cell proliferation (Figs. 6 A and B, *P*<0.05), while increased apoptotic cells by TUNEL staining and cleaved caspase-3 (Fig. 6 E) as well as cleaved PARP (Fig. 6 F).for indicated the effects of rapamycin apoptosis on the tumor cells (*P*<0.05).

## Discussion

Since the prevalence of ASCC is not as high as other cancers in the US, and some cases have rather good prognoses, there is little impetus to develop new therapeutic strategies to treat ASCC and bring them to a stage of clinical trials. There is an urgent need for development of a good animal model for ASCC that can aid in the exploration of experimental therapies. Here we have reported a novel anal cancer model in *Tgfbr1/Pten* 2cKO mice. Compared with previously reported anal cancer models [6], [15], [18], anal cancer in the present model is spontaneously generated without the use of a carcinogen such as DMBA, which may activate the ras family that is not widely found in human anal cancers. This animal model also displays rapid anal tumorigenesis, with an average time of 4–6 weeks as compared to 4–6 months in the previously reported animal model [6], [15], [18]. Moreover, the animal model shares more biological markers that are similar to other human cancers, such as activation of EGFR and increased levels of p-Stat3 and pro-inflammation cytokines, which could provide more strategies for exploring anal cancer treatments [16]. We believe this animal model will prove to be valuable preclinical animal model for development of chemopreventive therapies for SCC. However, this mouse model is in a mixed background and the induction of tumors by the compound deletion of Tgfbr1 and Pten may be dependent upon the genetic background. Therefore, further studies are necessary to ascertain the effects of a strain background on the susceptibility to tumorigenesis.

This mouse model has displayed cases of HNSCC (mostly tongue and buccal SCC) and ASCC, though each had a different penetrance, suggesting a close relationship between these two types of squamous cell carcinomas. The oral cavity and anus are the origin and the end of the digestive tract, respectively, and both are open to the environment, potentially causing continuous inflammation and an increase in the levels of pro-inflammatory cytokines induced by bacterial and viral infections. Our data have also shown increased mRNA level of pro-inflammatory cytokines such as Il1a, Il1b, Tnf, Ptgs2 in mouse ASCC as compared with control anal skin, which is well demonstrated in human and mice head and neck cancer [13]. Hoots et al. reported that 78% of squamous cell anal cancer cases involve some type of HPV infection, and that among them 66% are positive for the high-risk HPV16 genotype [14]. These viruses are intricately involved in anal carcinogenesis via the encoding oncoproteins E5, E6, and E7. Emerging evidence also supports the hypothesis that HPV infection contributes to the progression of HNSCC [19]. Additionally, the importance of Akt activation is also observed in both anal cancer and HNSCC [7], [20], although the issue of whether such a driver oncogenic pathway is caused by mutation of PIK3CA, loss of PTEN, HPV oncoproteins, or increased pro-inflammatory cytokines is still unknown [21]. The similarities between these two cancers may provide useful clues for further research on anal cancer.

We and others confirmed that the Akt/mTOR pathway is important to ASCC tumorigenesis with or without HPV infection [8], [14]. A study from the hospital of Wisconsin University has shown that the activation of mTOR was frequently detected in human anal cancers [14]. The preclinical mouse models also indicated that there was activation of mTOR in DMBA-induced anal cancers using HPV E6 and E7 transgenic mice [14]. Evidence from in vitro studies suggests that HPV E6 expression leads to increased activity of Akt/mTORC1 through the upstream kinases PDK1 and mTORC2 [3]. Consistent with these findings, we have also observed activation of Akt/mTOR in the non-HPV associated anal cancer, suggesting the essential role of mTOR in anal carcinogenesis in both HPV-associated and non-HPV-associated anal cancers. Our studies on ASCC and HNSCC demonstrate that our mouse model will prove to be valuable this to the value identifying combinatorial therapeutic regimens. These regimens include rapamycin- a putative mTOR inhibitors that could have greater success in treating human anal cancer, and could reduce instances of morbidity associated with the standards of treatment that are currently available. There is an accumulating evidence to indicate that genetic loss of PTEN in cell lines and animal will lead to activation of Akt/mTOR [22], [23]. It's interesting to know that PTEN deletion in mice by inducible K14-Cre will not develop anal SCC. While the loss of TGF-β signaling in the mouse epithelial or mesenchymal cells will have anal cancer with different penetration [12], [15].

Another interesting observation is that even in an era of antiretroviral treatment, men with AIDS have the highest incidence of anal cancer [16]. Furthermore, it has been reported that there is a 72% prevalence of high-risk HPV in anal swabs and a 43% prevalence of high-grade anal intraepithelial neoplasia (AIN) among HIV-positive men who have sex with men [13]. These data suggest that anal cancer may be related to immunodeficiency. Also of interest is the fact that our present model is based upon the knockout of TGF-β receptor I (*Tgfbr1*), which has proven to play a pivotal role in mobilization and recruitment of immune cells [12], [15]. The absence of *Tgfbr2* may also predispose the mice to genital and anal cancers [18]. Coincidentally, the HPV16 oncoprotein E7 could reduce the expression of TGFBR2 in mouse cervical tissues [24], suggesting the potential roles of TGF-β receptors in these HPV-related cancers. On the other hand, loss of TGF-β receptors may weaken TGF-β-induced apoptosis, enhance cytokine secretion, and in inflammatory bowel disease promote the papilloma transformation and tumor angiogenesis [10], [15], [18], [25]–[27]. Whether the loss of TGF-β signaling initiates molecule events or just promotes carcinogenesis initiated by HPV infection [6], [18], [28], it is obvious that TGF-β signaling plays an important role in the tumorigenesis of ASCC, and may prove to be an important therapeutic target. mTOR has important roles in tumorigenesis, inflammation, immunity, angiogenesis, and autophagy in both epithelia as well as mesenchymal environment [29]–[31]. Accumulating evidence indicates a pivot role of Akt/mTOR pathway in regulating CD4 + Foxp3+ regulatory T cells in tumor microenvironment [32], [33].Therefore, targeting mTOR by rapamycin may be a promising therapeutic strategy for ASCC.

In summary, we report here a novel anal cancer model with spontaneous and rapid tumorigenesis that will prove to be a valuable preclinical animal model suitable for developing effective therapeutic strategies to treat cancer. We have also demonstrated important role of the Akt/mTOR pathway in anal cancer progression, and its inhibition by rapamycin treatment results in delayed cancer progression indicating therapeutic potential of rapamycin in treating anal cancer.
